# EMERGENCY DEPARTMENT PRACTICE PATTERNS FOR OLDER ADULTS WITH ABDOMINAL PAIN

**DOI:** 10.1093/geroni/igad104.2983

**Published:** 2023-12-21

**Authors:** Rachel Wu, Ari Friedman

**Affiliations:** University of Pennsylvania, Philadelphia, Pennsylvania, United States; University of Pennsylvania, Philadelphia, Pennsylvania, United States

## Abstract

Abdominal pain is the most common reason for visit (RFV) to the emergency department (ED) for older adults in the United States. Despite high morbidity and mortality, unlike for chest pain, there is no standardized diagnostic pathway. We investigated the rates of computed tomography (CT) scans and X-rays for older adults and compared these to the rates in young and middle-aged adults. We conducted survey-weighted descriptive analyses of the National Hospital Ambulatory Medical Care Survey. We included 27,149 sampled ED chart abstractions for abdominal pain, representing 143.2 million visits nationally; 12.6% were from older adults 65+. CT rates increased over time for all age categories, with 26.2% of all patients receiving a CT scan in 2007 to 42.6% in 2019. The relative percent change in abdominal CT scans was greatest for older adults, with a 30.3% change from 2007 to 2019, compared to 24.0% change for adults aged 46-64 and 15.0% for adults aged 18-45. 13% of older adults received only an X-ray, which is neither sensitive nor specific for acute pathology in older adults in the ED. Test positivity, as defined by receiving an emergency general surgical diagnosis among patients imaged with CT or ultrasound increased from 17.2% in 2007 to 22.9% in years 2019 (p< 0.01), suggesting increasing severity for this high-risk condition among older adults. Our findings suggest potential over-use of ineffective testing (X-rays) as well as the need for better targeted cross-sectional imaging (rising test positivity).

